# To Be or Not to Be in Thrall to the March of Smart Products

**DOI:** 10.1002/mar.20920

**Published:** 2016-09-08

**Authors:** Fiona Schweitzer, Ellis A. Van den Hende

**Affiliations:** ^1^ University of Applied Sciences Upper Austria; ^2^ Delft University of Technology

## Abstract

This article explores how perceived disempowerment impacts the intention to adopt smart autonomous products. Empirically, the paper builds on three studies to show this impact. Study 1 explores the relevance of the perceived disempowerment in respect of smart autonomous products. Study 2 manipulates autonomy of smart products and finds that perceived disempowerment mediates the link between smart products’ autonomy and adoption intention. Study 3 indicates that an intervention design―that is, a product design that allows consumers to intervene in the actions of an autonomous smart product―can reduce their perceived disempowerment in respect of autonomous smart products. Further, Study 3 reveals that personal innovativeness moderates the role that an intervention design plays in product adoption: an intervention design shows a positive effect on adoption intention for individuals with low personal innovativeness, but for those with high personal innovativeness no effect of an intervention design is present on adoption intention. The authors suggest that managers consider consumers’ perceived disempowerment when designing autonomous smart products, because (1) perceived disempowerment reduces adoption and (2) when targeted at consumers with low personal innovativeness, an intervention design reduces their perceived disempowerment.

Google presented its first prototype of an autonomous car without a steering wheel, brake pedal, clutch or accelerator in the spring of 2014. Similarly, European car manufacturers and research institutions have been developing autonomous driving systems for years, and test runs on European and American streets have just begun (Waters & Sharman, 2015). Autonomous cars are one among many smart products that might change future consumption dramatically. Industry insiders expect machine‐to‐machine (M2M) communication, a guiding technology behind smart products, to grow from the current 10 billion devices to 50 billion in 2020, resulting in a bountiful new wave of smart autonomous solutions for industry and consumers (Evans, 2011; Gershenfeld, Krikorian, & Cohen, 2004).

Smart products are products that contain information technology. They can either need human interaction to function (semiautonomous smart products), or work fully autonomously (autonomous smart products). Users operate semiautonomous smart products, for example, via the smart phone, to have the product perform a certain task. Autonomous smart products can operate and interact with other devices without human interaction; they often also independently improve their performance by means of learning algorithms (Gershenfeld et al., 2004; Porter & Heppelmann, 2014). For example, smart heating systems are semiautonomous smart products when they are connected to a smart phone, which enables their users to switch heating on or off from remote places. The consumer has to install the heating system, set the schedule, adjust features, and control it from his or her smart phone app as necessary. When smart heating systems adapt the home temperature after a data exchange with home owners’ digital calendars, incorporate learning algorithms enabling them to learn about the residents’ heating habits and, by automatically adapting the heating behavior, optimize the energy efficiency and comfort, they function as autonomous smart products.

Not only do autonomous smart products take tasks over from the consumer and perform them independently, but consumer input is also no longer needed for this task (Rijsdijk & Hultink, 2003, 2009). For example, autopilots land airplanes independently, and sophisticated autonomous smart lawn mowers start mowing when the grass exceeds a certain length without user interaction. Since no consumer input is needed, users may perceive autonomous smart products as comfort‐increasing devices that increase task fulfillment's efficiency and effectiveness, or liberate consumers from unwanted chores (Cronin, 2010). At the same time, consumers can also feel disempowered. They feel that they lose influence on how and when a task is performed (Heiskanen et al., 2007; Tenner, 2003). For example, autonomous cars without steering wheels and brake pedals provide users with very limited possibilities to intervene if the car malfunctions or hackers attack its IT systems.

Perceived disempowerment is (potential) users’ feeling that an innovation reduces their freedom to choose or act, and causes them to lose control and autonomy (Heiskanen et al., 2007; Johnson, Bardhi, & Dunn, 2008; Rijsdijk & Hultink, 2003).

Several studies point out a negative effect of perceived disempowerment on adoption (e.g., Heiskanen et al., 2007; Mick & Fournier, 1998). Yet, this evidence is largely anecdotal or based on qualitative study insights. This paper seeks to remedy this shortcoming by investigating the link between perceived disempowerment and adoption in an experimental setting. Presuming that perceived disempowerment reduces adoption, the paper further aims to complement previous work by investigating the extent to which managers can reduce consumers’ perceived disempowerment in respect of autonomous smart products when they include features that allow them to interfere with autonomous products’ actions.

The paper proceeds as follows: first, the theory and the hypotheses are presented. Second, three complementary studies investigating the relevance of perceived disempowerment in the context of smart products are described. The first study presents qualitative insights into the issue. The second study uses different music apps as stimuli and investigates the impact of perceived disempowerment on the adoption intention. In the third study, the negative effect of consumers’ perceived disempowerment on adoption is replicated in a smart home context. The same study also shows how, by making simple changes in a product design, practitioners can help reduce autonomous smart products’ disempowering effect by allowing users to interfere. The third study also examines the effect of users’ technological innovativeness on the disempowerment‐adoption link. The article concludes with a discussion of the perceived disempowerment effect on the adoption of autonomous smart products for marketing practice and research.

## CONCEPTUAL BACKGROUND AND HYPOTHESES

The psychological empowerment literature (Giddens, 1984; Spreitzer, 1996; Thomas & Velthouse, 1990) understands empowerment individuals’ willingness and perceived ability to exert control and influence over decisions that affect their life, and to shape their environment, or a specific environmental context.

Thomas and Velthouse (1990) identify four elements of empowerment: meaningfulness, self‐efficacy, self‐determination, and impact. Meaningfulness is the value that individuals attribute to an action or an object based on their ideals and norms. If individuals consider an environmental context as meaningful, they engage in it, while disengagement, apathy, and resignation are the consequences of interpreting a situation as being not meaningful. Meaningfulness corresponds to the user involvement construct, a psychological state reflecting an object's personal relevance (Barki & Hartwick, 1994).

Self‐efficacy is the belief in one's capabilities to master a situation, or produce a desired outcome, through one's actions (Bandura, 1977; Block & Keller, 1997). In the context of technology adoption, high self‐efficacy signifies that consumers perceive themselves as being able to handle and use a certain technological product based on their set of skills, knowledge, and abilities, as well as on their prior experience with other products (Compeau, Higgins, & Huff, 1999; Thatcher & Perrewe, 2002). Self‐determination describes whether individuals have the choice to initiate, regulate, and carry out an action presupposing a wish to act (Deci & Ryan, 1987; Ryan & Deci, 2000). Self‐determination is absent when environmental contexts force individuals to act in a certain way, while self‐determination is present when they can choose a certain action (Elie‐Dit‐Cosaque, Pallud, & Kalika, 2012). Impact describes the degree to which individuals perceive their participation in a task making a difference. Put differently, impact mirrors a consumer's perceived ability to impact the outcome of an activity in a certain context. The concept is a context‐specific derivation of the locus of control concept, which is a global personality trait (Wolfe & Robertshaw, 1982). When the perceived impact is low, individuals feel powerless, when it is high, they feel able to produce an expected outcome (Conger & Kanungo, 1988).

Management research has focused on the effect of perceived empowerment on work performance and has found that perceived empowerment increases managerial effectiveness and innovative behavior (e.g., Spreitzer, 1996), but decreases turnover intentions (Ertürk & Vurgun, 2014). Technology adoption research has studied the elements of perceived empowerment separately and has identified perceived meaningfulness (Amoako‐Gyampah, 2007; Barki & Hartwick, 1994), self‐efficacy (Shu, Tu, & Wang, 2011), and self‐determination (Morris, Hall, Davis, Davis, & Walton, 2003) as important drivers of technology adoption.

A few consumer studies investigate perceived empowerment as an aggregate, holistic concept. Taylor and colleagues (1992) understand consumers as empowered when they are able to choose between alternative market offerings. A person's perception of having a choice contributes to the positive emotional experience of a situation (Hui & Bateson, 1991; Meuter, Bitner, Ostrom, & Brown, 2005). Choice and the feeling of self‐governance put individuals in control, increase their enjoyment of a task, and provide the freedom to evaluate and select a certain alternative, rather than being assigned one, while a lack of choice makes them feel undermined (Dahl & Moreau, 2007; Murray & Häubl, 2011). Individuals thus react negatively when their freedom of choice is constrained (Brehm, 1989; Fitzsimons & Lehmann, 2004).

Fuchs and colleagues (Fuchs & Schreier, 2011; Fuchs, Prandelli, & Schreier, 2010) extend empowerment beyond choice in the purchase situation; they understand user empowerment as the control users have over the corporate new product development process. For example, consumers can have a say in developing products by cocreating a specific product's design, or by coselecting the design to be produced, for example, the T‐shirt production of the company Threadless. Each week, Threadless receives approximately 500 T‐shirt designs from its user community, who then votes for their favorite T‐shirts. Threadless produces the five most favored T‐shirts and pays the consumers who designed them. Fuchs et al. (2010) show that consumers who coselect products for a company to manufacture experience psychological ownership of such products, resulting in positive feelings and increased demand. Fuchs and Schreier (2011) find that not only customers who participate in cocreation, but also customers who do not actively participate themselves in the cocreation activity favor products from a company that involves other customers over one that does not.

Extrapolating from research, the same link between empowerment and adoption is likely to be present in the context of autonomous products, but one that works in the opposite direction. Perceived disempowerment is understood as oppositional in nature to perceived empowerment. This signifies that at one end of the empowerment scale, perceived empowerment is high and individuals perceive that they exert control and influence over decisions. On the other end of the scale, perceived disempowerment is high, that is, people feel not capable to control or influence a decision. Perceived disempowerment is similar to dependency. While products that are perceived as empowering provoke positive attitudes and adoption interest (Fuchs & Schreier, 2011), individuals that feel disempowered, feel they are unable to perform a certain task themselves and feel they depend on someone or something else for task realization. In this vein, technology dependency is understood as lack of freedom to decide independently how a task is performed and handing over this task to the machine (Fournier & Mick, 1999; Mick & Fournier, 1998). Heiskanen et al. (2007) indicate that products that are perceived as disempowering provoke negative attitudes and reduce the adoption intention. Johnson et al. (2008) demonstrate that feeling in control and the perceived freedom to act is not only the prerequisites for trust in technology, but also increases product satisfaction. Consumers who perceive autonomous smart products as disempowering, fear that such products may function more in the interest of a corporation than in the consumer's interest (Heiskanen et al., 2007; Tenner, 2003), may take actions they are not intended to take (Bechtold & Sotoudeh, 2013), or make decisions that users would rather make themselves (Heiskanen et al., 2007). Users are thus reluctant to use these products; a case in point is the MS Word autocorrect function, which many MS Word users switch off, because it makes unwanted changes to their text (Rijsdijk & Hultink, 2003). These arguments lead to the following hypothesis:

**H1**: Consumers’ perceived disempowerment in respect of autonomous smart products decreases product adoption.

With the expansion of smart products that work autonomously, the decision competence is shifting from individuals toward the machine (Decker, 2014). Autonomous smart products that independently take over various tasks and decisions (Rijsdijk & Hultink, 2003) obscure technical processes and reduce the user's ability to intervene. With reduced possibilities to interfere, users may perceive autonomous smart products as patronizing, feeling that the product makes decisions that they prefer to make and reduces their freedom and choice (Fournier & Mick, 1999; Heiskanen et al., 2007). The opaque functioning of autonomous smart products may make it difficult for users to understand the way autonomous smart products act and why they act in a specific way. Autonomous smart products can then become a source of mistrust (Mick & Fournier, 1998).

Products that deprive individuals of their freedom of action and choice provoke psychological reactance (Murray & Häubl, 2011; Wicklund, Clee, & Wicklund, 1980). Individuals who perceive themselves as lacking freedom try to reestablish their freedom by opposing the object or individual limiting this (Brehm, 1966, 1989; Wicklund et al., 1980). To reduce psychological reactance and increase adoption, companies can provide interfaces in autonomous smart products that increase consumers’ freedom to intervene and choose. For example, an autopilot that lands a plane independently, usually also allows an airline pilot to easily take over control and land the plane, if the pilot decides so. The autopilot design thus includes an empowering element that allows the user to intervene. The user can enjoy the benefits of the autonomous process as long as the user wishes to, but as soon as he or she feels better off taking over control, the user is free to do so. Such a design can reduce mistrust in technology and relieve from fear of malfunctioning and the inability to intervene in case of malfunctioning (Covey, Link, & Merrill, 2012; Cronin, 2010).

Individuals do not have to understand each step of a computer algorithm, but the human–machine interface should allow basic possibilities of intervention (Lee & See, 2004; Murray & Häubl, 2011). For example, the interface could show the user what the system is planning to learn, explain what the expected result of this learning process will be, and ask the user for permission to carry out this learning step. Such an interface might reduce autonomous smart products’ efficiency (Behmann & Wu, 2015), but it will allow the users to intervene, putting them back in control, reducing their perceived disempowerment in respect to autonomous smart products, and increasing adoption. Following the above reasoning, it is hypothesized:

**H2**: An intervention design (i.e., interface design that allows the user to intervene) reduces perceived disempowerment, in turn increasing the adoption of autonomous smart products.

The authors further suppose that personal innovativeness influences perceived disempowerment's effect on adoption. In the technology domain, personal innovativeness describes an individual's tendency to adopt technological innovations before others do (Agarwal & Prasad, 1998; Goldsmith & Hofacker, 1991).

Regarding autonomous smart products, Rijsdijk and Hultink (2003) theorize that consumers vary considerably in their perception of these products’ advantages and risks. Personal innovativeness may explain these individual differences. Individuals with higher innovativeness are open to change (Steenkamp, Hofstede, & Wedel, 1999), are inclined to take the risk of adopting new products (Rogers, 2005), gather more information on technical products (Hirunyawipada & Paswan, 2006), possess more technical products (Goldsmith, Freiden, & Eastman, 1995), and perceive them as more useful than individuals with lower innovativeness do (Yi, Fiedler, & Park, 2006). Their increased interaction with technical products reinforces innovative users’ positive attitude toward them and leads them to trusting these products (Fang, Singh, & Ahluwalia, 2007; Johnson et al., 2008; Lewis & Weigert, 1985). Consequently, innovative consumers may pay more attention to the advantages that autonomous technical products offer, and fear them not working in the intended way, or not benefitting the user, far less (Hirunyawipada & Paswan, 2006). Perceived disempowerment is less of an issue for innovative individuals, because of their high trust in technology. This trust makes them feel confident that the product will perform well (Bruner & Kumar, 2007; Lee & See, 2004) and, thus, more inclined to adopt a new technological product (Gefen, 2004). Stated more formally, the following is proposed:

**H3**: Personal innovativeness moderates the effect of an intervention design on perceived disempowerment such that high personal innovativeness attenuates the positive effect of an intervention design on perceived disempowerment.

## STUDY 1

### Purpose

The goal of this qualitative study is to examine whether perceived disempowerment is an issue that customers identify as relevant in the context of smart autonomous products.

### Method

Data were collected from 134 representatives of local households. The sample consisted of 54.5% women with a median age class of 40–44 (on an answer scheme ranging from 20 to 79 in constant age classes). The participants were invited to a lab on the university campus and asked to evaluate a new product concept. This concept described a health monitor that records and displays eating habits, work‐out, and health data. The device can take blood samples and provides information on cholesterol levels, blood sugar, and nutrient balance. An external chest strap sensor measures calorie consumption when exercising and automatically transferred the data to the Health monitor. With the blood pressure wristband, it also measures blood pressure data that is wirelessly transferred to the Health Monitor. It can also register daily food intake and makes suggestions about groceries and recipes to reach dietary targets in a healthy and sustainable way. The device suggests options to respond to medical data, such as high cholesterol values, and has a feature that suggests which groceries should be bought. It automatically sends the data to a doctor, who analyzes the data and provides online consultation.

After reading through the concept description, participants had five minutes to write down what they perceived as the advantages and disadvantages of the product.

### Analysis and Results

The analysis of participants’ responses followed a qualitative analysis procedure (Miles & Huberman, 1994). The coders identified each respondent's individually perceived advantages and disadvantages through identifiers such as bullet points, semicolons, commas, or periods. In an open coding procedure, a coder screened roughly 20% of the full data set of advantages and disadvantages that the participants provided to identify in vivo codes from the raw data. Based on this prescreening, the coder developed a preliminary codebook of category codes (Auerbach & Silverstein, 2003). Thereafter, two additional coders reviewed all the statements independently and coded them with this codebook, adding codes where appropriate. Afterwards, the derived codes were compared and, in case of disagreement, a third coder determined the final coding. All three coders were blind to the goals of this study.

The list in the Appendix presents the full list of category codes with a definition of the category codes and illustrative examples of the participants’ original statements. The participants enumerated 197 statements on the perceived disadvantages of the product and 280 advantages. Perceived disempowerment was one category of disadvantages that the participants mentioned most often (73 statements representing 37% of all statements regarding the disadvantages). The majority of these statements (56) focused on technology dependence and the danger of losing the ability and possibility to independently assess what a healthy lifestyle is. The respondents generally perceived a “too big dependence on the specifications and settings of the device regarding nutrition,” or simply “technology dependence.” A smaller number of statements (17) expressed fear that the product may reduce one's willingness to take individual responsibility. For example, a participant wrote: “stultification because you no longer think for yourself.” These findings suggest the relevance of perceived disempowerment in respect of smart autonomous products and encourage the study of its impact on adoption behavior.

## STUDY 2

### Purpose

The purpose of Study 2 is to investigate whether perceived disempowerment reduces the intention to adopt autonomous smart products (H1). Study 2 uses two stimuli (semiautonomous and autonomous smart product) and investigates their impact on adoption. Further it includes perceived disempowerment as a mediator of this impact.

### Method

#### Participants and Design

A total of 55 undergraduate and graduate students were willing to participate in the experiment with participation in a raffle for 1 of 10 €10 gift vouchers from a local supermarket as a potential reward. One participant was excluded for providing incomplete data. Of the remaining 54 students, 52.7% were women. The students participated in a between‐subjects experiment (stimuli: semiautonomous vs. autonomous smart product).

#### Procedure, Stimuli, and Measures

The participants were given a product description, which differed in the two conditions, to read. In the semiautonomous condition, the potential product user could select different music for download. In the autonomous condition, the music app automatically selected music for download based on self‐learning algorithms fed by past user behavior. Both concepts were smart phone apps. Table 1 shows the wording of the stimuli.

**Table 1:** Stimuli of Study 2

Stimulus	Stimulus Description
Semiautonomous stimulus	You are just listening to music you like on a radio, or in a pub, and you do not know the band. Simply draw a clef in the air with your hands and the app searches for the title of the song and the name of the band. You can listen to it, read information on the song and the band, and buy and download the song in an online music shop by clicking on it on your smartphone.
Autonomous stimulus	This app enables you to easily find new songs that suit your music taste. The app automatically downloads five new songs to your smartphone each month. Depending on the songs that you currently have on your smartphone, how often you listen to which songs, and how you rate songs online (e.g., on YouTube), the app automatically downloads new songs for you

To investigate H1, the effect of the stimuli on adoption intention and perceived disempowerment as a mediating variable is studied. To control for alternative explanations to perceived disempowerment, participants were asked to evaluate perceived newness. This control variable was included, because it is possible that autonomous products are perceived as newer and more innovative than semiautonomous products and may demand new behavior of the user (Hoeffler, 2003; Rijsdijk & Hultink, 2003, 2009). For example, semiautonomous parking assistance systems that assist the driver get into a parking space through signals indicating distance to walls, sidewalks, or other cars are serialized in new cars and do not change the actions the driver has to perform to get into the space. Autonomous parking assistance are currently only available as pilots and demand drivers to get out of the car, push a button on their smart phone, and keep it pressed while the car moves independently forth and back until it is in the parking spot.

The participants completed measures of product adoption, perceived newness, and perceived disempowerment. For product adoption, an intention scale was used (“Now I would like you to think about how much you would like to have this app”; 1 = definitely not like to have; 7 = definitely like to have). The scale for perceived disempowerment was adapted from Meuter et al. (2005) and included the following four items: “This app makes decisions that I would prefer to make myself,” “I fear that this app could take actions that I dislike,” “This app reduces my possibilities to decide to which music I want to listen,” and “This app reduces my freedom of action.” (7‐Point scale, ranging from 1 = do not agree at all to 7 = fully agree, *α* = 0.86.)

The participants responded to a three‐item perceived newness scale to measure product newness (Hoeffler, 2003) anchored by 1 = not very innovative, not very novel, and not very original and 7 = very innovative, very novel, and very original, *α* = 0.88).

### Analysis and Results

#### Direct Effects

In an ANOVA, the participants in the autonomous condition reported lower adoption intention than in the semiautonomous condition (*M*_autonomous condition_ = 2.97, SD = 1.50 vs. *M*_semiautonmous condition_ = 3.88, SD = 1.42; *F*(1,53) = 5.40, *p* = 0.024). Further, participants in the autonomous condition showed higher perceived disempowerment than participants in the semiautonomous condition (*M*_autonomous condition_ = 4.24, SD = 1.59 vs. *M*_semiautonmous condition_ = 2.18, SD = 1.18, *F*(1,53) = 29.33, *p* < 0.001). Regarding perceived newness (M_autonomous condition_ = 4.26, SD = 1.73 vs. *M*_semiautonmous condition_ = 4.46, SD = 1.80; *F*(1, 53) = 0.17, *p* = 0.681), participants in the two conditions did not differ.

#### Mediation

A mediation analysis was carried out to test the hypothesis following the procedure suggested by Preacher and Hayes (2008). The model included adoption intention as the dependent measure, with smart product autonomy (semiautonomous = 0, autonomous = 1) as the independent measure, and perceived disempowerment as the mediator.

First, the total effect of the independent variable on adoption was significant (*F* 1,53) = 5.40, *β* = −0.92, *t* = −2.32, *p* = 0.024). Second, the effect of the smart product autonomy stimuli on perceived disempowerment was significant (*F*(1, 53) = 29.33; *β* = 2.06; *t* = 5.42, *p* < 0.001). Third, the model regressing the independent variable and the mediator on adoption intention was significant (*F* (2, 52) = 13.10, *p* < 0.001). The direct effect of perceived disempowerment was significant (*β* = −0.54, *t* = −4.35, *p* < 0.001) and the direct effect of the stimuli on adoption was insignificant (*β* = 0.19, *t* = 0.44, *p* = 0.66), indicating full mediation. The indirect effect through the mediator was significant with a point estimate of −1.11 and a 95% bias corrected confidence interval of −1.08 to −0.39. Sobel's test also supported the mediating effect (*z* = −3.36; *p* < 0.001). Overall, mediation results provided evidence for H1.

### Discussion

The findings of Study 2 highlight the importance of perceived disempowerment in respect of smart autonomous products. A student sample of smart phone users was asked to evaluate two apps. The respondents were less likely to adopt the autonomous apps than the semiautonomous apps. Mediation analysis revealed the mediating role of perceived disempowerment; respondents perceived the autonomous app as more disempowering and perceived disempowerment decreased the intention to adopt the app. The finding supports the hypothesis that perceived disempowerment in respect of autonomous smart products decreases product adoption.

## STUDY 3

### Purpose

Study 3 has three objectives. First, the study tests whether an interface design that allows the user to intervene increases the intention to adopt smart autonomous products (H2). Second, it strives to replicate the effect of perceived disempowerment on product adoption (H1). Third, it investigates whether perceived disempowerment's impact on smart product adoption intent differs according to a consumer's personal innovativeness (H3).

### Method

#### Participants and Design

The 274 participants were randomly assigned to one of the two manipulated intervention design conditions (with/without an intervention design). Personal innovativeness served as a measured independent variable in this study. Participants were representative of the general employable population and were recruited via a market research agency. Their average age was 44 years, their monthly net income €2200, and 53.3% were women.

#### Procedure, Stimuli, and Measures

The experiment had a concept test setting. Participants were asked to participate in a test of the attractiveness of a new product concept and received the concept description to read and then answer questions on how interesting this product would be for them. They subsequently received one of two product stimuli. Both stimuli represented a smart home application with the same introductory explanation. (“This software for computers, smartphones, or tablets works with any heating system and regulates your heating autonomously. The software knows when you are at home and when you leave home and functions accordingly.”) In the autonomous condition without intervention design, participants received an autonomous stimulus without any elements that might reduce the perceived disempowerment. (“This software learns about your heating needs and automatically adapts your home heating based on your online calendar (e.g. on MS Outlook), the weather forecast, and your heating habits. For example, this software learns that you leave home at 8 a.m. on workdays and automatically reduces the heating to 17 degree. In the evening, when you are 20 minutes from home, it automatically starts to increase the temperature to 21 degree. In this way, the software optimizes the temperature at home and contributes to comfortable living and the saving of energy.”)

In the autonomous condition with intervention design, participants received a smart autonomous product stimulus with the potential product disempowerment reduced by means of a product design element that gives users the possibility to actively influence the smart product's activities. The exact wording of the stimulus was as follows: “This software tells you which specific ‘actions’ it wants to automatize based on your online calendar (e.g. on MS Outlook), the weather forecast, and your heating habits. For example, it suggests: ‘Planning to decrease temperature to 17 degree after 8 a.m. on weekdays and turning up heating again in the evening to 21 degree when you are 20 minutes away from home.’ You can press the ‘OK’ button to endorse this action, or press ‘decline’ to refuse it. In this way, the software optimizes the temperature at home and contributes to comfortable living and the saving of energy.”

In constructing the stimuli, attention was paid to developing stimuli varying in intervention design, but equal regarding autonomy, newness, and understandability. To check the manipulation, the participants answered questions regarding their perceived ability to intervene (intervention possibility: “I think that it would be easy for me to intervene if the product does not act in the way I want it to,” 1 = not agree at all, 7 = fully agree), *M* = 4.45, SD = 1.58) as well as regarding autonomy, newness, and understandability of the presented stimuli. For newness (“not very innovative”/“very innovative,” “not very novel”/“very novel,” “not very original”/“very original,”; *α* = 0.92) and understandability (“not very understandable”/“very understandable,” “not very clear”/“very clear,” “not very uncomplicated”/“very uncomplicated,”; *α* = 0.96) Hoeffler's scales (2003) were adopted. For autonomy (“This product goes its own way,” “This product takes the initiative,” “This product works independently,” and “This product does things by itself”; *α* = 0.91) the Rijsdijk and Hultink (2009) scale was used. All scales were measured from 1 = low newness, understandability, and autonomy to 7 = high newness, understandability, and autonomy.

After reading the product concept description, the participants indicated adoption intention. (“How much would you like to have this heating software?” 1 = definitely not like, 7 = definitely like, *M* = 3.59, SD = 1.75). For perceived disempowerment the same scale as in Study 2 (*α* = 0.91) is used. Personal innovativeness (Agarwal & Karahanna, 2000) is measured with three items (“If I heard about a new information technology, I would look for ways to experiment with it,” “Among my peers, I am usually the first to try out new information technologies,” “I like to experiment with new information technologies”; 7‐point scale (1 = not agree at all, 7 = fully agree, *α* = 0.92).

### Analysis and Results

#### Manipulation Check

A mean comparison between the ratings of the condition without (*M* = 4.21, SD = 1.53) and with intervention design (*M* = 4.69, SD = 1.60) delivered significant results (*F*(1, 273) = 6.25, *p* = 0.013) for the perceived ability to intervene. Subjects who evaluated the autonomous solutions with intervention design, perceived higher intervention possibilities than those who received the autonomous stimulus without intervention design. In accordance with expectations, the stimuli in the condition without intervention design (*M* = 4.74, SD = 1.52) and with intervention design (*M* = 5.03, SD = 1.31, *F*(1,273) = 3.04, *p* = 0.082) were neither significantly different regarding newness, nor autonomy (no intervention design: *M* = 5.16, SD = 1.24; intervention design: *M* = 4.84, SD = 1.37, *F*(1,273) = 3.85, *p* = 0.051), or understandability (no intervention design: *M* = 5.19, SD = 1.47; intervention design: *M* = 5.40, SD = 1.30 *F*(1,273) = 1.51, *p* = 0.22), confirming that the manipulation was successful. Further, the respondents in both groups were not significantly different regarding personal innovativeness (no intervention design: *M* = 3.796, SD = 1.691; intervention design: *M* = 3.440, SD = 1.799, *F*(1,273) = 2.832, *p* = 0.094).

#### Findings

A moderated mediation analysis with product adoption as the dependent variable, the two design stimuli as the independent variable (0 = no intervention design, 1 = intervention design) perceived disempowerment as a mediator, and personal innovativeness as a moderator of the link between stimuli and perceived disempowerment was carried out.

H3 predicts that the indirect effect of perceived disempowerment for the autonomy–adoption intention relationship would be attenuated by high personal innovativeness. To assess moderated mediation (Preacher & Hayes, 2008; Preacher, Rucker, & Hayes, 2007), four conditions were examined.

First, the relationship between the stimulus condition (no intervention design/with intervention design) and adoption intention were investigated with the moderator and mediator absent demonstrating a significant and positive effect (*F*(1, 272) = 19.13, *β* = 0.89, *t* = 4.37, *p* < 0.001). Conforming with H2, the analysis demonstrated that the respondents had a higher adoption intention regarding the autonomous solution with individual intervention possibilities (*M* = 4.04, SD = 1.77) than when the design did not provide such possibilities (*M* = 3.15, SD = 1.61). This finding indicates that the stimulus with intervention design was preferred to the stimulus without intervention design, supporting H2.

Second, the mediating role of perceived disempowerment was studied. The effect of the stimuli on perceived disempowerment is negative and significant (*F*(1, 272) = 28.92, *β* = −0.93, *t* = −5.38, *p* < 0.001). Also, the effect of perceived disempowerment on adoption intention is negative and significant (*F*(2,271) = 42.55, *β* = −0.51, *t* = −7.86, *p* < 0.001)). The total effect model for mediation demonstrates that the total effect of the independent variable on adoption intention consists of a direct effect of 0.42 (*t* = 2.16, *p* = 0.03) and an indirect effect of 0.47 (with a 95% confidence interval of 0.29 to 0.72). Sobel's normal theory test underlines mediation (*β* = 0.47, *z* = 4.41, *p* < 0.001). In conformity with H1, the results imply a mediating effect of perceived disempowerment in so far as higher perceived disempowerment reduces adoption intention and the designs with intervention design reduces perceived disempowerment compared to the design without intervention design.

Third, stimulus condition × personal innovativeness is significant and has a positive effect on perceived disempowerment (*F*(3, 270) = 28.99, *β* = 0.42, *t* = 4.55, *p* < 0.001). In the same model, stimulus condition has a negative and significant effect (*β* = −2.53, *t* = −6.88, *p* < 0.001) and personal innovativeness also has a negative and significant effect on perceived disempowerment (*β* = −0.47, *t* = −7.19, *p* < 0.001).

Fourth, the effects of intervention design on adoption intention via perceived disempowerment differ across low and high levels of personal innovativeness. Using Preacher and Hayes’ (2008) bootstrapping methodology, the index of moderated mediation is −0.21 (95% confidence interval: −0.33 to −0.11). The conditional indirect effects of intervention design on adoption intention via perceived disempowerment are positive and significant for individuals low in personal innovativeness (*point estimate* = 0.89; 95%, *confidence interval*: 0.61 and 1.19), but not significant for individuals high in personal innovativeness (*point estimate* = 0.15, *95*% *confidence interval*: −0.11 and 0.46). These results support H3.

**Figure 1:**
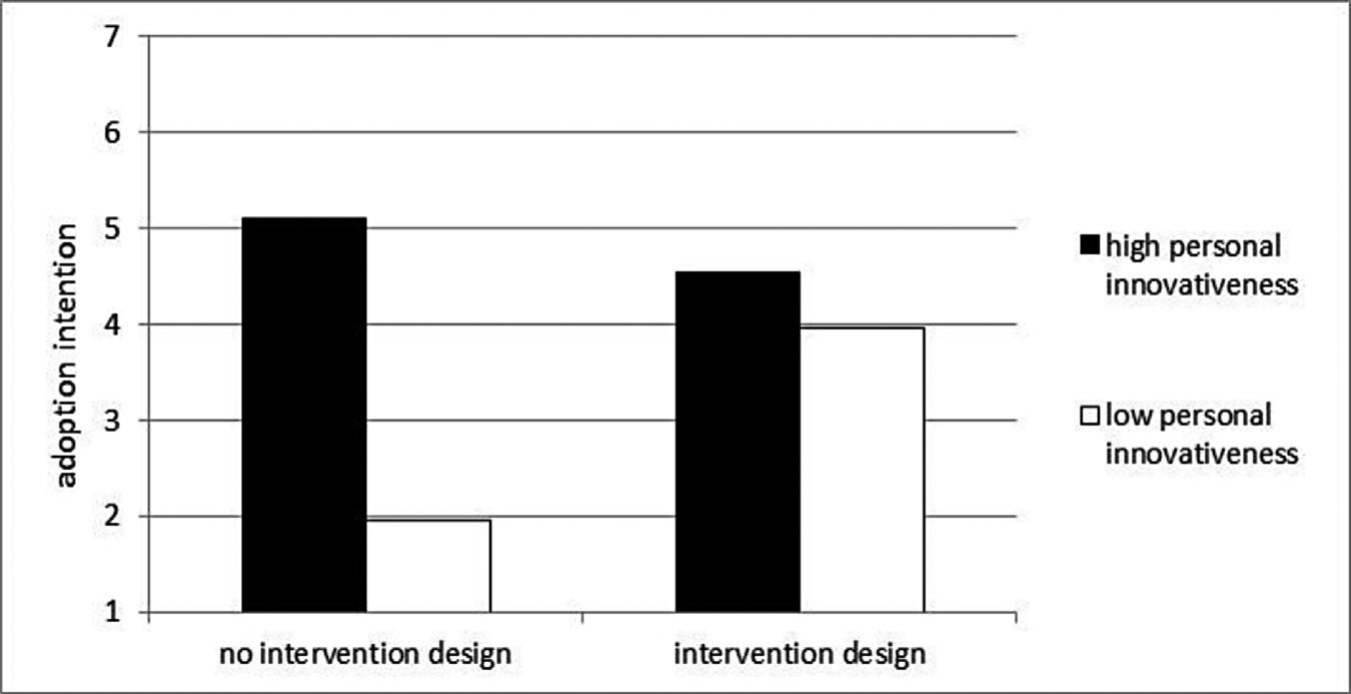
Product adoption as a function of intervention design and personal innovativeness.

Figure 1 graphically depicts the statistically significant interactions between intervention design and personal innovativeness. To draw Figure 1, a simple slopes test (Cohen & Cohen, 1983) was conducted. Simple slopes for respondents with higher personal innovativeness (+ 1 SD above the mean) and respondents with lower personal innovativeness (−1 SD below the mean) were estimated to illustrate the nature of interactions. The figure demonstrates the moderating role of personal innovativeness on the link between intervention design and adoption intention: On the one hand, intervention design has a strong effect on adoption intention if personal innovativeness is low and increases adoption intention. On the other hand, intervention design plays a minor role if personal innovativeness is high. In the latter case, intervention design slightly reduces adoption intention.

### Discussion

The findings of Study 3 indicate that respondents perceive autonomous smart products with intervention possibilities as less disempowering and are more inclined to adopt them than similar products without such possibilities. Put differently, the reluctance to adopt autonomous smart products can be reduced by means of a sophisticated product design that reduces the products’ perceived disempowerment (an intervention design).

The findings also demonstrate that personal innovativeness may be a critical segmentation variable when designing autonomous smart products. Individuals with low personal innovativeness have a strong preference for products with an intervention design. The two product design stimuli did not differ significantly in respect of participants with higher levels of personal innovativeness. Contrary to the effect that an intervention design has on individuals with low personal innovativeness, those with high personal innovativeness tend to prefer automated smart products without an intervention design.

## GENERAL DISCUSSION

### Theoretical Implications

To the authors’ knowledge, this is the first paper in the consumer research literature to explicitly postulate, or to empirically test, the effect of perceived disempowerment on smart product adoption. By providing insights into the interaction between perceived disempowerment and smart product adoption, this paper contributes to the understanding of the potential driver of users’ reluctance to adopt autonomous smart products.

The paper builds on three empirical studies to test the effect of perceived disempowerment on autonomous smart product adoption. Study 1 demonstrates that individuals associate smart autonomous products with disempowerment. Study 2 shows that consumers perceive autonomous smart products as more disempowering than semiautonomous smart products, and that this affects the adoption intention negatively. Study 3 finds that an intervention design reduces the perceived disempowerment of autonomous smart products and increases the intention to adopt them. Further, the study finds that individuals with a low degree of personal innovativeness prefer intervention possibilities, while this is not true for individuals with a high degree of personal innovativeness.

According to the seminal work by Geoffrey (1999), there is a chasm between innovators and early adopters on the one side (characterized by high personal innovativeness), and the majority and laggards on the other side (characterized by low personal innovativeness). Geoffrey (1999) highlights the importance of understanding the different needs of the consumers on the two sides as a means to bridge the chasm. Smart solutions with differing types of designs to reduce perceived disempowerment may well be such needs. Individuals with low personal innovativeness need an intervention design that will permit them to allow or refuse actions that an autonomous smart product will learn. Individuals with high personal innovativeness have more trust in technology and thus feel more at ease with autonomous smart products that do not offer such intervention possibilities. They might find interface designs with intervention possibilities burdensome.

### Managerial Implications

This paper's findings imply that product designs that allow consumers to intervene in autonomous smart products’ actions may be an effective feature in new product positioning strategies. Positioning strategies aiming at consumers with a high degree of innovativeness may focus on autonomous product solutions without intervention possibilities. Managers may offer product options with an intervention design when positioning strategies targeting consumers with a low degree of personal innovativeness.

### Limitations and Further Research

Although this paper takes an important initial step to examine the effect of perceived empowerment on the adoption of autonomous smart products, this research is not without limitations. These limitations offer opportunities for further research.

First, the studies focus on music apps and smart home software. These products are both low investment experience goods. Consumers may respond differently to autonomous smart products characterized by a high investment. There may also be differences between search and experience goods. Future studies could therefore investigate whether the effect of perceived disempowerment on adoption holds true for other types of autonomous smart products (e.g., autonomous cars).

Second, Study 3 investigates an intervention design that asks users to allow or decline an automated product's intended learning task before the product actually learns it. This is only a single solution to decrease perceived disempowerment. Future studies may investigate other product design strategies that decrease perceived disempowerment and increase adoption intention. Intervention designs provide consumers with the possibility to make choices, yet consumers may find it difficult to predict their degree of satisfaction with future outcomes (Kahneman, 1994). Exploration of the impact of different product designs with differing numbers of choice options (Wathieu et al., 2002) would enrich the understanding of how firms should design autonomous smart products to reduce perceived disempowerment while maximizing adoption.

Third, the research finds that personal innovativeness has a moderating effect on the relationship between an intervention design and adoption intention, yet only provides a single explanatory mediator. Identifying additional mediating variables that explain why individuals with low and high innovativeness differ regarding their intentions to adopt autonomous products with and without intervention design is an interesting avenue for further research. The authors hope that this research stimulates and inspires further research on the role of perceived disempowerment in the context of autonomous smart products.

## List of Perceived Advantages and Disadvantages of a Smart Autonomous Product

**Table:**

Category	Definition	Example	Number of Times Mentioned
Perceived Product Disadvantages			
Surveillance	Users fear that a third person can access data and use it to his/her benefit	“Third persons can control what you do”; “Who has access to my health data? I do not want the cashier in the supermarket to tell me to put back the bacon”	24
Disempowerment	Users feel dependent on technology and fear losing autonomy and control of their activities	“Contributes to patient immaturity”; “You lose the ability to evaluate yourself what is good for you”; “Decreases your autonomy thorough shopping lists and permanent updates for your doctor”; “You only see groceries from the participating supermarkets; the product indirectly directs your consumption behavior”	73
Usability	Users think that the product is difficult to handle and use	“It is difficult to pick it up and take it with you”; “The many elements of the product—external chest strap, blood pressure wristband—will make it difficult to handle it correctly”	34
Negative emotions	Users fear that negative emotions will arise every time you use the product	“Using the product intensifies the feeling of being ill”; “bad conscience”; “you no longer dare eat sweets because you want to avoid ‘living in sin’”	33
Danger for unstable persons	Users feel that the product might endanger mentally impaired persons	“The product is not useful for hypochondriacs or people with anxiety disorders”	2
No substitute	Users do not perceive the product as a useful substitute for consulting a doctor	“You still need to visit a doctor”	15
Time	Users expect the product to lead to users and/or doctors expending much time	“Medico needs time slots during his working hours for data analysis”; “very time‐consuming”	8
Cost	Users fear the financial burden of buying and using the product	“In the beginning, this product will certainly be very expensive and only top athletes will be able to afford it”	8
Sum of enumerated product disadvantages			197
Perceived product advantages			
Transparency	Users’ habits and health condition will be transparent	“Makes nutrition intake transparent, including food calories, fat in food, etc.”; “You have a better overview and knowledge of the personal data that is relevant when exercising.”	104
Reaction	Users expect to be able to react fast to the medical data	“Allows you to react fast in case of illness”	17
Health awareness	Users expect the product to increase their motivation to remain healthy and their health awareness	“I am certain that it improves health awareness”; “It encourages healthy eating”; “It helps you understand the consequences of your eating habits”	40
Risk awareness	Users expect the product to increase their risk awareness	“Helps detect health risks”	14
Health status	Users expect the product to improve their health status	“The product improves your attitude to life, because it helps you feel good and increases your performance”	14
Usability	Users believe the product is easy to use	“Handling seems to be pretty straightforward”	18
Achievement of personal targets	Users believe that the product helps achieve personal dietary targets	“It will help reduce weight”; “Makes you more disciplined regarding losing weight and helps to do so in a healthy way”	25
Multifunctionality	Users like the product's multifunctionality	“It combines different approaches for living healthily”; “All in one—many different features in one product”	6
Information	Users appreciate receiving useful information regarding how to change behavior (e.g., recipes for a healthy diet)	“The product delivers shopping tips and recipes”; “The shopping button ‘on sale’ is very useful”	15
Comfort	Users enjoy the comfort of monitoring their health status at home and fewer doctor consultations	“You can check your health comfortably from home, no need to make an appointment with the doctor”	20
Help in daily life	Users see the benefit of assistance in their daily life	“Precise information on health nutrition and exercise”; “Good support in everyday life”	6
Independence	Users feel that the product increases their ability to live an individual and independent life	“Makes you self‐reliant, independent of a doctor—your health is in your hands”	1
Sum of enumerated product advantages			280
